# An Improved Random Walker with Bayes Model for Volumetric Medical Image Segmentation

**DOI:** 10.1155/2017/6506049

**Published:** 2017-10-23

**Authors:** Chunhua Dong, Xiangyan Zeng, Lanfen Lin, Hongjie Hu, Xianhua Han, Masoud Naghedolfeizi, Dawit Aberra, Yen-Wei Chen

**Affiliations:** ^1^Department of Mathematics and Computer Science, Fort Valley State University, Fort Valley, GA, USA; ^2^College of Computer Science and Technology, Zhejiang University, Hangzhou, China; ^3^Radiology Department, Sir Run Run Shaw Hospital, Medical School of Zhejiang University, Hangzhou, China; ^4^Graduate School of Information Science and Engineering, Ritsumeikan University, Kyoto, Japan

## Abstract

Random walk (RW) method has been widely used to segment the organ in the volumetric medical image. However, it leads to a very large-scale graph due to a number of nodes equal to a voxel number and inaccurate segmentation because of the unavailability of appropriate initial seed point setting. In addition, the classical RW algorithm was designed for a user to mark a few pixels with an arbitrary number of labels, regardless of the intensity and shape information of the organ. Hence, we propose a prior knowledge-based Bayes random walk framework to segment the volumetric medical image in a slice-by-slice manner. Our strategy is to employ the previous segmented slice to obtain the shape and intensity knowledge of the target organ for the adjacent slice. According to the prior knowledge, the object/background seed points can be dynamically updated for the adjacent slice by combining the narrow band threshold (NBT) method and the organ model with a Gaussian process. Finally, a high-quality image segmentation result can be automatically achieved using Bayes RW algorithm. Comparing our method with conventional RW and state-of-the-art interactive segmentation methods, our results show an improvement in the accuracy for liver segmentation (*p* < 0.001).

## 1. Introduction

Segmentation of organ from CT volume is an important prerequisite for computer-aided surgery, computer-assisted intervention, and image-guided surgery. The accurate segmentation of the organ from clinical CT images is considered a challenging task: Large variations in shape make an accurate segmentation difficult, and existing lesions (e.g., tumors) exhibit considerable variation for the organ anatomical structure. To accurately segment an organ, various approaches have been proposed in literatures [1–8], such as intensity-based [9–11], classification-based [12, 13], clustering-based [14–18], statistical shape model- (SSM-) based [19, 20], probabilistic atlas- (PA-) based [21–25], active contour- (AC-) based [26, 27], and watershed-based [28, 29] segmentation methods. However, the main challenge of the abovementioned methods is the fast and efficient segmentation of large image data. This can be observed particularly in medical applications where a resolution of three-dimensional CT and MRI body scans constantly increases.

Recently, a growing interest is attracted by an interactive graph-based image segmentation algorithms such as graph cut (GC) [30–36] and random walker (RW) [37–41] algorithms. The random walker algorithm represents a recent noteworthy development in the weighted graph-based interactive segmentation methods. This technique with user interaction is more suitable for volumetric medical images to guarantee the reliability, accuracy, and fast speed demands.

However, due to the classical RW algorithm definitions on the weighted graphs, for a high-resolution volumetric medical image, RW method needs to construct the corresponding large-scale graph to solve the resulting sparse linear system, which leads to high computation cost: the long computation time and the high memory usage. Hence, over the past years, a large amount of research has been conducted to extend and enhance the random walker algorithm. Grady et al. [40] extended the classical RW segmentation approach by combining the regional intensity priors. The sparse linear equations can be addressed by the preconditioned conjugate gradient to achieve an acceptable memory consumption and easy parallelization. In [41], the computational demands with RW are alleviated by introducing an “offline” precomputation before user interaction with RW in real-time “online.” Using a similar principle, an offline precomputation was used to further speed up the online segmentation in [42]. Both methods used the “offline” and “online” strategies to minimize the time spent waiting. In addition, Goclawski et al. [43] proposed a superpixel-based random walker method to reduce the graph size, while the computation time increases linearly with the number of superpixels. The accuracy of superpixels plays an immediate decisive role in the process of organ segmentation.

To resolve these limitations, in our previous research [44], we proposed a knowledge-based segmentation framework for the volumetric medical image in a slice-by-slice manner based on the classical random walker. This algorithm employs the previous segmented slice as the prior knowledge for automatically setting the object/background seed points for the adjacent slices. It can reduce the graph scale and significantly speed up the optimization procedure of the graph. However, the classical RW algorithm was designed to be a general purpose interactive segmentation method, such that a user could mark a few pixels with an arbitrary number of labels and expect a quality result, regardless of the data set or the segmentation goal. Segmentation of a medical image ignores itself absolute intensity and shape information. If a consistent intensity and shape profile characterize an object of interest, then this information should be incorporated into the RW segmentation process.

Taking these into consideration, in our study, we extended a classical random walker algorithm by incorporating the prior (shape and intensity) knowledge in the optimization of sparse linear system. The objective of our work is to combine the prior knowledge with the spatial cohesion of the random walker algorithm in a principled way that produces the correct result. Based on the extended random walker, we applied a knowledge-based segmentation framework for the volumetric medical image in a slice-by-slice manner. Our strategy is to employ the previous segmented slice to obtain the prior (shape and intensity) knowledge of the target organ for the adjacent slice. With a small number of user-defined seed points, we can obtain the segmentation results of the start slice in the volume which can be used as the prior knowledge of the target organ. According to this prior knowledge, the object/background seed points are automatically defined and the corresponding Bayes model can be generated. Integrating this Bayes model into the RW sparse system, the organ is automatically segmented for the adjacent slice.

The remainder of this paper is organized as follows.  Section 2 presents a brief recapitulation of the random walker algorithm and then extends to incorporate the prior (shape and intensity) knowledge.  Section 3 elaborates our proposed knowledge-based framework using the extended RW with the Bayes model.  Section 4 contains experimental work, and Section 5 discusses the implementation of our method, followed by the conclusion (Section 6).

## 2. Development

The random walk algorithm treats image segmentation as an optimization problem on a weighted graph, where each node represents a pixel or voxel. Therefore, we firstly define the graph that we are working on. We use the following notations for the rest of the paper. Given an image, *I*, a graph consists of *G* = (*V*, *E*) with vertices (nodes) *v* ∈ *V* and edges *e* ∈ *E*. Each node *v*_*i*_ in *V* uniquely identifies an image pixel *x*_*i*_. An edge, *e*, spanning two vertices *v*_*i*_ and *v*_*j*_, is denoted by *e*_*ij*_. A weighted graph assigns a weight to each edge. The weight of an edge, *e*_*ij*_, is denoted by *w*_*ij*_. It represents the similarity between two neighboring nodes *v*_*i*_ and *v*_*j*_. The degree of a vertex is *d*_*i*_ = ∑_*j*_*w*_*ij*_ for all edges *e*_*ij*_ incident on *v*_*i*_.

### 2.1. Review of Random Walker Method

The random walker segmentation algorithm of [37] computes the probability, for each pixel, that a random walker leaving that pixel will first arrive at a foreground seed before arriving at a background seed. It was shown in [37] that these probabilities may be calculated analytically by solving a linear system of equations with the graph Laplacian matrix. The Laplacian matrix is defined as

(1) $$\begin{array}{c} L_{ij}=\left\{\begin{array}{ll} d_{i} & i=j\\ -w_{ij} & v_{i}\text{ and }v_{j}\;are\;adjacent\;nodes\\ 0 & otherwise, \end{array}\right. \end{array}$$

where *L*_*ij*_ is indexed by vertices *v*_*i*_ and *v*_*j*_. *w*_*ij*_ = exp(−*β*(*I*_*i*_ − *I*_*j*_)^2^) is the edge weight, and *I*_*i*_ and *I*_*j*_ indicate the image intensity at vertices *v*_*i*_ and *v*_*j*_, respectively. *β* represents a tuning constant that depends on the user.

Given a weighted graph, a set of marked (labeled) nodes, *V*_*M*_, and a set of unmarked nodes, *V*_*U*_, such that *V*_*M*_ ∪ *V*_*U*_ = *V* and *V*_*M*_∩*V*_*U*_ = ∅, we would like to label each node *v*_*i*_ ∈ *V*_*U*_ with a label *s*. *s* = 1 stands for the foreground, and *s* = 2 stands for the background. Assuming that each node *v*_*j*_ ∈ *V*_*M*_ has also been assigned with a label *s*, we can compute the probabilities, *x*_*i*_^*s*^, that a random walker leaving node *v*_*i*_ arrives at a marked node *v*_*j*_ by solving the minimization of

(2) $$\begin{array}{c} E_{internal}^{s}=\frac{1}{2}x^{sT}{Lx}^{s}. \end{array}$$

All nodes *V* are divided into two sets: the marked (prelabeled) nodes *V*_*M*_ and unlabeled (i.e., free) nodes *V*_*U*_. Therefore, the above function can be reformulated as follows:

(3) $$\begin{array}{c} E_{internal}^{s}=\frac{1}{2}\left[x_{M}^{sT}x_{U}^{sT}\right]\left[\begin{array}{cc} L_{M} & B\\ B^{T} & L_{U} \end{array}\right]\left[\begin{array}{c} x_{M}^{s}\\ x_{U}^{s} \end{array}\right]. \end{array}$$

Minimization of (3) with respect to *x*_*U*_^*s*^, the random walker problem can be solved by the following system of equations:

(4) $$\begin{array}{c} L_{U}x_{U}^{s}=-B^{T}x_{M}^{s}. \end{array}$$

The variable *x*_*U*_^*s*^ represents the set of probabilities corresponding to unmarked nodes; *x*_*M*_^*s*^ is the set of probabilities corresponding to marked nodes (i.e., “1” for foreground nodes and “0” for background nodes). By virtue of *x*_*i*_ being a probability,

(5) $$\begin{array}{c} \overset{2}{\underset{s=1}{\sum }}x_{i}^{s}=1\;\forall i. \end{array}$$

The random walk algorithm is explained in detail elsewhere [37]. Next, we will now present how the incorporation of the Bayes model into the above framework yields a segmentation algorithm.

### 2.2. Random Walker with Bayes Model

According to the above priori knowledge, we can calculate a posterior probability *p*(*s*|*I*_*i*_) at the node *v*_*i*_ which belongs to the label *s*. Assuming that each label is equally likely, Bayes theorem gives the probability that a node *v*_*i*_ belongs to label *s* as

(6) $$\begin{array}{c} x_{i}^{s}=\frac{p\left(s\left|I_{i}\right.\right)}{\sum_{q=1}^{2}p\left(q\left|I_{i}\right.\right)}=\frac{p\left(I_{i}\left|s\right.\right)\times p\left(s\right)}{\sum_{q=1}^{2}p\left(I_{i}\left|q\right.\right)\times p\left(q\right)}, \end{array}$$

where *p*(*I*|*s*) is the likelihood map for an organ and *p*(*s*) is the shape map for the targeted organ. *p*(*s*) can be obtained by dilating the targeted organ region in the previous segmented slice. *p*(*I*|*s*) can be estimated by the previous segmented slice of the organ. *s* = 1 is the foreground, and *s* = 2 is the background.

Equation (6) can be also written in vector notation:

(7) $$\begin{array}{c} \left(\overset{2}{\underset{q=1}{\sum }}\Lambda^{q}\right)x^{s}=p\left(s\left|I\right.\right), \end{array}$$

where *Λ*^*s*^ is a diagonal matrix with the values of *p*(*s*|*I*) on the diagonal.

According to (6), the minimum energy distribution for the external function is

(8) $$\begin{array}{c} E_{external}^{s}=\overset{2}{\underset{q=1,q\neq s}{\sum }}x^{qT}\Lambda^{q}x^{q}+{\left(x^{s}-1\right)}^{T}\Lambda^{s}\left(x^{s}-1\right). \end{array}$$

To incorporate the posteriori probability function (external term) into the RW algorithm (internal term), we may optimize the following energy:

(9) $$\begin{array}{c} E_{total}^{s}=E_{internal}^{s}+\gamma E_{external}^{s}. \end{array}$$

The first term is the driving force behind the spatial cohesion of the random walker algorithm. The second term is a Bayes penalty term with the weight *γ* used to guarantee robustness to small disconnected pieces. The used Bayes model is generated according to the prior knowledge of an organ: shape and intensity. In this work, we set the weight to *γ* = 0.01.

The minimum energy of the above equation is obtained when *x*_*s*_ satisfies the solution to

(10) $$\begin{array}{c} \left(L+\gamma \overset{2}{\underset{q=1}{\sum }}\Lambda^{q}\right)x^{s}=\gamma p\left(s\left|I\right.\right). \end{array}$$

Optimizing this energy leads to the system of linear equations:

(11) $$\begin{array}{c} \left(L_{U}+\gamma \overset{2}{\underset{q=1}{\sum }}\Lambda^{q}\right)x_{U}^{s}=\gamma p\left(s\left|I_{U}\right.\right)-B^{T}x_{M}. \end{array}$$

The usage of the proposed Bayes-based RW algorithm is strongly limited by the enormous size of the graph represented in 3D volumetric medical image and the necessity of solving a huge sparse linear system. It results in the relative increase of the unlabeled seed points relative to a 2D image. Hence, in order to estimate the probability of each unlabeled seed point, the extended RW algorithm needs to calculate the larger inverse matrix (*L*_*U*_ + *γ*∑_*q*=1_^2^*Λ*^*q*^)^−1^, which leads to high computation costs: long computation time and high memory usage. We integrated our extended RW algorithm into a knowledge-based framework to make it more suitable and workable for our application. The following details our knowledge-based framework and results.

## 3. Knowledge-Based Framework

**Figure 1:**
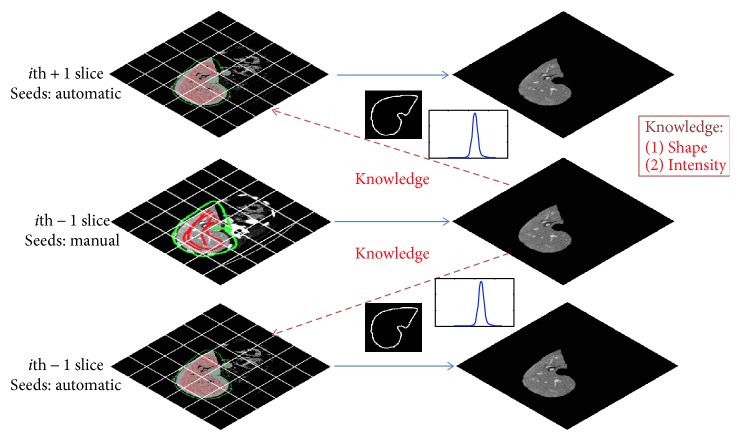
The whole procedure of our knowledge-based method.

Our knowledge-based strategy employs the previous segmented slice as the prior (shape and intensity) knowledge of the target organ for automatic segmentation of the adjacent slice. Using a small number of user-defined seed points, we can obtain the segmentation results of the start slice of the volume for use as the prior knowledge of the target organ. According to the prior knowledge, the object/background seed points can be dynamically updated for the adjacent slice by combining the narrow band threshold (NBT) method and the organ model with a Gaussian process. Meanwhile, the corresponding Bayes model can be generated. Finally, an extended Bayes-based random walker algorithm is applied to automatically segment the whole volume in a slice-by-slice manner. In our work, “object” means the target organ to be segmented and “background” means the other tissues except the target organ. The whole procedure of the proposed approach is shown in Figure 1. In this method, there is a three-step pipeline consisting of the following:

1. Selecting and segmenting the start slice, as shown in the middle-part of Figure 1: (a) Manually defining the object/background seed points. (b) Generating a Gaussian model (GM) using the seed points. (c) Segmenting the organ (“Candicate Pixels” for the liver) using the classical RW method.
2. Segmenting the adjacent slice, as shown in the upper-part and bottom-part of Figure 1: (a) Generating a Gaussian model (GM) according to the previous segmented organ (intensity knowledge). (b) Automatic setting the object/background seeds based on the restricted region by morphological operation of the previous segmented organ (shape knowledge). (c) Refining the seed points based on NBT. (d) Segmenting the organ using our proposed Bayes-based RW methods. Thus, it automatically segments the whole organ in the remaining slices based on the updated prior knowledge of the organ.
3. Smoothing the boundary of the whole volume: Finally, the boundary of the output volume is smoothed by “Fourier transform” that forms the final organ surface.

In the following section, we will introduce the start slice segmentation, the GM generation, and automatic seed point selection which integrate the prior intensity and shape knowledge of the previous segmented organ.

### 3.1. Interactive Segmentation of the Start Slice

Our proposed segmentation is a slice-by-slice method. There are two main steps in our proposed method. The first step is to segment the start slices interactively, and the second step is to segment other remaining slices automatically based on the segmented start slices. The aim of the first step (interactive segmentation of the start slices) is to find the initial region of the target organ (liver) so that it can be used as prior (intensity and shape) knowledge of the organ as the following steps for automatic segmentation.

**Figure 2:**
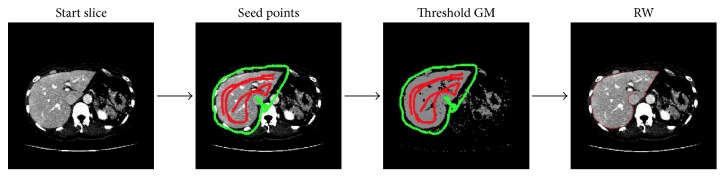
Interactive segmentation of the start slice in CT image.

The process of the first interactive segmentation of two start slices is shown in Figure 2 and involves four steps: (1) manually select one axial start slice. Scanning an input CT volume along the axial axis to find one slice in which the organ has the relative larger cross section in the axial plane; (2) manually define the object/background seeds on this start slice; (3) automatically generate the thresholded images based on the constructed Gaussian model (GM) using these seeds. To remove the intercostal muscles and the other nonobject parts, the object seeds are employed to construct the approximate intensity models for this organ using the Gaussian model (GM). After estimating the statistical intensity model, the constructed model is thresholded to find “Candidate Pixels” for the organ; (4) automatically segment the thresholded images. The final step of this process is to segment the thresholded image based on “Candidate Pixels” by the classical RW method.

### 3.2. Automatic Segmentation of the Adjacent Slice

#### 3.2.1. GM for Generation of the Thresholded Image

Constructing a Gaussian model (GM) [45] is aimed to estimate a new preprocessed image of the target organ so that it can more easily distinguish the difference between the target organ and other tissues. As explained in the last section, the initial segmented slice can be used to estimate the statistical parameters of the liver model for the current slice. Due to the existence of a large number of the liver pixels, estimation of the statistical parameters can be trusted. A Gaussian model is employed to estimate the intensity distribution of the liver. The Gaussian model is given by

(12) $$\begin{array}{c} p\left(I_{i}\left|s\right.\right)=\frac{1}{\sqrt{2\pi \sigma_{s}^{2}}}\exp\left(\frac{-{\left(I_{i}-\mu_{s}\right)}^{2}}{2\sigma_{s}^{2}}\right),\\ p\left(I_{i}\left|s\right.\right)=\frac{p\left(I_{i}\left|s\right.\right)}{\sum_{q=1}^{2}p\left(I_{i}\left|q\right.\right)}, \end{array}$$

where the parameters mean *μ*_*s*_ and variance *σ*_*s*_^2^ can be estimated by the marked seed points or the previous segmented slice of the organ. *I*_*i*_ indicates the image intensity at the node *v*_*i*_. *s* = 1 is the object, and *s* = 2 is the background.

**Figure 3:**
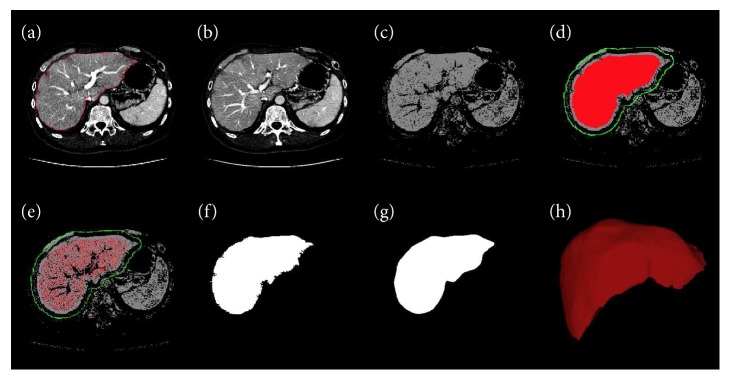
Steps of the RWBayes method. (a) The segmented liver (red) of the previous slice; (b) the current slice; (c) candidate pixel by thresholding the GM; (d) the rough object (red) and background (green) seed points; (e) the fine seed points using a NBT method; (f) the initial segmentation result by RWBayes; (g) smoothing the boundary by Fourier transform; and (h) visualisation of the segmented liver volume.

The intensity models are automatically determined for each slice according to the segmented organ in the previous slice. Furthermore, in order to remove some nonobject parts and obtain an accurate result, we threshold the output of this intensity model by discarding probabilities less than 0.5, so it can generate a likelihood map of the object. Comparison of the original CT image (Figure 3(b)) with the corresponding intensity model (Figure 3(c)) revealed that the liver can be more easily distinguished from other tissues. However, for the background, the likelihood map keeps the original probability value without thresholding.

#### 3.2.2. Automatic Setting of Seed Points

The main assumption in our method is that it can determine the approximate prior (shape and intensity) knowledge for the organ. Due to a slice-by-slice technique that is applied to segment the organ in our method, the user segments one slice in the volume to define this prior knowledge, and consequently, they are automatically updated for the nearby slices. In this approach, assuming the consequent slices of the same patient have a high correlation, the boundary of the organ in the next slice does not go far from its border in the previous slice. Thus, a defined shape constraints based on the previous slice can be used to roughly select the object/background seed points for the adjacent slice.

Assuming the cross-section of the liver in the *i*th slice is divided into *m* parts and the region of the organ for each part (Mask_*i*,*j*_, 1 ≤ *j* ≤ *m*) is known, corresponding to the part *j* in the (*i* + 1)th slice, the object and background seeds can be defined by the following equation:

(13) $$\begin{array}{c} {BS}_{i+1,j}=\left({Mask}_{i,j}⊕{BE}_{Dilation2}\right)-\left({Mask}_{i,j}⊕{BE}_{Dilation1}\right),\\ {FS}_{i+1,j}={Mask}_{i,j}⊕{RE}_{Erosion}, \end{array}$$

where Mask_*i*,*j*_ is the mask of the organ corresponding to the *j*th part in the slice *i*. BE_Dilation1_ and BE_Dilation2_ are the structuring elements used for dilation in the region. RE_Erosion_ is the structuring elements used for erosion in the region. These elements are empirically selected to be disks with a radius of BE_Dilation1_ = 10 pixels, BE_Dilation2_ = 8 pixels, and BE_Erosion_ = 8 pixels.

The background seed points are directly selected in the current slice in the region BS_*i*+1,*j*_ which can be considered as accurately seeded points outside the liver's boundary. However, as shown in Figure 3(d), it can be seen that there were still a lot of false positives (other tissues) in the FS_*i*+1,*j*_ despite eroding the liver region for the previous slice, because we cannot segment the previous slice accurately and there still exists a variation of liver shape for different slices.

#### 3.2.3. Refinement of Seed Points

As already explained above, it can dynamically update the parameters of GM model for the following slices. If the intensity model of the liver includes the parameters *μ* and *σ*, we can threshold this component in the narrow region [TL, TH] to find the fine seed points corresponding to the candidate liver pixel.

(14) $$\begin{array}{c} TL=\mu -\beta \sigma ,\\ TH=\mu +\beta \sigma . \end{array}$$

We empirically found that the values of *β* are in the range [0.05, 0.3] corresponding to low-contrast and high-contrast datasets.

In addition, it can been seen from Figure 3(d), since the defined region FS_*i*+1,*j*_ may include the nonliver part (such as vessels); we can threshold the narrow band to achieve more accurate object seeds (Figure 3(e)). Thus, for a pixel located in the region FS_*i*+1,*j*_, if the intensity value of this pixel belongs to the narrow range [TL, TH], it is considered as an object seed. After estimating the “Candidate Pixels” and the fine object/background seeds for the current slice, the Bayes-based RW algorithm is applied to segment the liver (Figure 3(f)).

### 3.3. Smoothing the Boundary of the Whole Volume

However, the boundary of the segmented object obtained in the last step is not smooth, as shown in Figure 3(f). If the coordinates of the boundary points are analyzed by the Fourier transform (FT), they contain a significant number of high-frequency components. According to the definition of the FT, the coordinates (*x*, *y*) are transformed from the spatial domain into the frequency domain as

(15) $$\begin{array}{c} \begin{array}{cc} \begin{array}{l} F_{x}\left(k\right)=\overset{N}{\underset{j=1}{\sum }}x\left(j\right)e^{\left(-\frac{2\pi i}{N}\left(j-1\right)\left(k-1\right)\right)}\\ F_{y}\left(k\right)=\overset{N}{\underset{j=1}{\sum }}y\left(j\right)e^{\left(-\frac{2\pi i}{N}\left(j-1\right)\left(k-1\right)\right)} \end{array} & i=\sqrt{-1} \end{array}, \end{array}$$

where *N* is the number of the boundary points that are usually greater than 100. The boundary is smoothed by removing the high-frequency components, while the useful (information bearing) low-frequency components are retained. Hence, the first 15 components in frequency domain are kept and then transferred into the spatial domain (Figure 3(g)).

(16) $$\begin{array}{c} \begin{array}{cc} \begin{array}{l} x\left(j\right)=\overset{15}{\underset{k=1}{\sum }}F_{x}\left(k\right)e^{\left(\frac{2\pi i}{N}\left(j-1\right)\left(k-1\right)\right)}\\ y\left(j\right)=\overset{15}{\underset{k=1}{\sum }}F_{y}\left(k\right)e^{\left(\frac{2\pi i}{N}\left(j-1\right)\left(k-1\right)\right)} \end{array} & i=\sqrt{-1} \end{array}. \end{array}$$

## 4. Results

### 4.1. Database

Our dataset included 26 CT images of the abdominal region with a resolution of 0.683 × 0.683 × 1 mm^3^ and a size of 512 × 512 × (159–263) pixels. All of the data were stored in DICOM image format with a depth of 12 bits per pixel. These data were acquired by GE LightSpeed Ultra scanners with eight detectors. The large variation of liver images was an important feature in the evaluation of our segmentation method. Hence, data were acquired from normal and pathological cases between 20 and 75 years old. The sample contained 20 normal cases and 6 pathological cases: no. 1 to no. 20 were normal cases and no. 21 to no. 26 belonged to pathological cases. Therein, patients (pathological cases) were those who were suspected of having a disease, such as chronic liver disease, and were scanned in the course of diagnosis. In order to make a quantitative evaluation for our proposed method, the liver was segmented for each image (i.e., subject) manually as the ground truth. The segmentation was performed under the guidance of a physician in order to obtain accurate liver volumes. This study was conducted with the approval of the institutional review boards at University Ethics Committee, and all data provided written informed consent.

The proposed algorithm was implemented in a MC-OS-based personal computer (Intel®Corei7 2.5GHz and 16GB-DRAM). The programming environment was coded in the MATLAB environment. Visualization of the shapes was performed using VTK [46] in C++ languages.

### 4.2. Quantitative Measurement

To measure the accuracy of our method, we compared it with the conventional RW method and the state-of-the-art interactive segmentation algorithms by two metrics.

#### 4.2.1. Dice Coefficient (Dice)

The dice coefficient is one of the most popular methods to evaluate segmentation accuracy. This metric is given in percent and based on the voxels of two binary 3D volumes, with *V*_manual_ as the manually and *V*_auto_ as the automatically segmented organs.

(17) $$\begin{array}{c} Dice=\frac{2\left|V_{manual}\cap V_{auto}\right|}{\left|V_{manual}\right|+\left|V_{auto}\right|}\times 100\%. \end{array}$$

#### 4.2.2. Volumetric Overlap Error (VOE)

The volumetric overlap error between two sets of voxels *V*_manual_ and *V*_auto_ is given in percent. This ratio is also known as Tanimoto or Jaccard coefficient.

(18) $$\begin{array}{c} VOE=\frac{\left|V_{manual}\cap V_{auto}\right|}{\left|V_{manual}\cup V_{auto}\right|}\times 100\%. \end{array}$$

### 4.3. Quantitative Validation of Liver Segmentation

**Figure 4:**
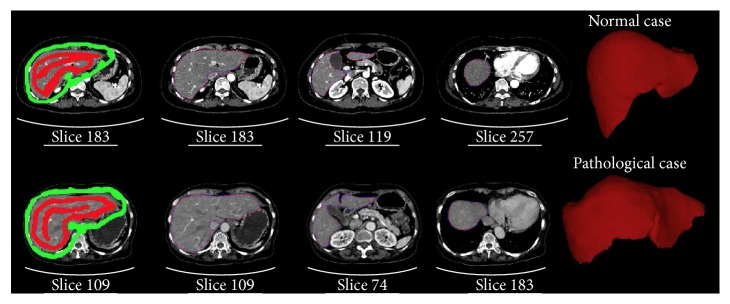
Comparison of the manual segmentation (blue) with the segmentation results of our method (red). The first row is the segmentation result in case 9. The second row is the segmentation result of pathological case with the unusual liver shape in case 22.

To investigate the performance of our proposed segmentation method, we applied our proposed RWBayes method to 26 clinical CT volumes which are described in the previous section. The segmentation results of two typical cases are shown in Figure 4. The results in Figure 4 proved that performing the RWBayes method to segment the livers can give us accurate results. A common difficulty for computer-aided liver segmentation is the erroneous inclusion of heart volumes, which our method robustly avoided. It confirmed the ability of our method to segment the livers with a precision segmentation result.

Additional challenges come from enlarged livers, where the liver has large shape variations which made it very difficult to be segmented. Taking this limitation into consideration, in this research, our technique performed on 26 CT scans that combined normal cases and pathological cases with large morphological variations. Figure 4 shows the liver segmentation result from one pathological case. It proved the performance of our proposed algorithm which was robust for segmenting the liver in the pathological cases with large morphological variations.

**Figure 5:**
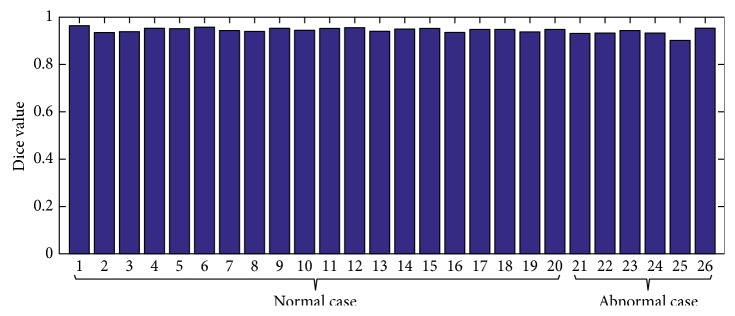
Our technique performed on 26 CT scans with Dice measurement. The first 20 data points are normal cases, and the remaining 6 data points are pathological cases.

Apart from a visual inspection, a quantitative evaluation was conducted.  Figure 5 gave a more clear depiction of the corresponding accurate results of 26 cases. The first 20 data points correspond to normal cases (the average Dice is 0.946), and the remaining 6 data points are pathological cases (the average Dice is 0.930). Regarding the result of applying our method to synthetic shapes, we can conclude that our proposed method was robust in addressing the segmentation of the liver (with the average Dice's similarity coefficient = 0.942). Future research directions will include applying our method on more datasets in order to more accurately evaluate the performance.

### 4.4. Qualitative Comparison of Interactive Segmentation Methods

To evaluate the effectiveness of the proposed method (RWBayes), RWBayes was compared with the classical random walk (RW3D) [37]. Considering the memory usage demands for applying the RW3D algorithm to the computer, we resized all of our datasets (512 × 512 × (159–263) pixels) into the size of 128 × 128 × 36 pixels. Moreover, we also compared our proposed method with a knowledge-based framework using the classical random walker and narrow band threshold (RWNBT) [44], in which the RWNBT did not generate a thresholded image based on the constructed Gaussian mixture model according to the previous segmented liver.

**Figure 6:**
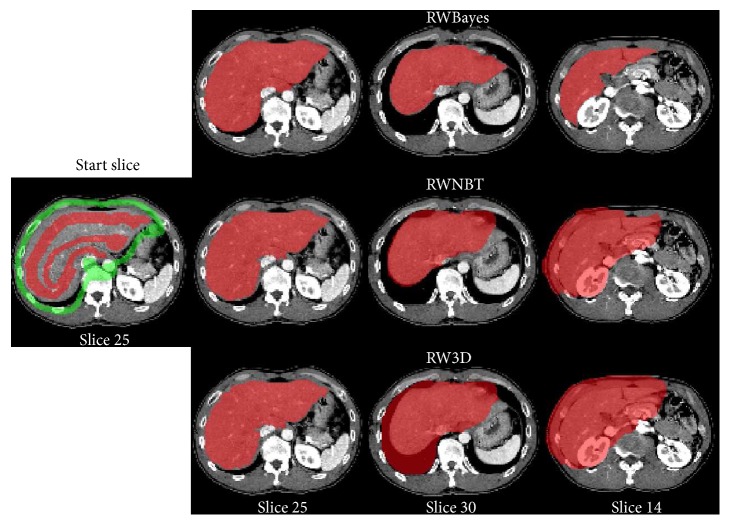
Comparison of the liver segmentation results with RWBayes method, RWNBT method, and RW3D method in case 6.

Quantitative and comparative results from applying the RW3D, RWNBT, and RWBayes methods for the liver segmentation are presented in Figure 6. In order to intuitively make a comparison between our proposed RWBayes and RW3D methods, it was unreasonable to give only one start slice with the corresponding segmentation result. It was necessary to show different slices for one data corresponding to a point on the curve with 128 × 86 × 33 pixels. The red images were segmented liver slices, which were overlaid with the original CT slices. The simulation verifies that the performance of RWBayes was significantly better than the RW3D and RWNBT methods for segmenting the liver.

In order to make a comparison with the state-of-the-art interactive segmentation algorithms, we also compared the results using the graph cut algorithm (GC) [34] and interactive K-means algorithm (IKM) [14].  Table 1 clearly depicts the merits of our method by listing the comparative results with the average of Dice, VOE, and runtime between automated and manual segmentations for all 26 test CT scans. Computation time is an important metric for evaluating one segmentation algorithm. For the classical RW algorithm, the basis of RW method is a large, sparsely occupied linear equations, whose size corresponds to the number of voxels in the 3D image. Hence, it exhibited slowness for solving 3D image segmentation. A significant reduction in runtime values using RWBayes-based segmentation compared with those based on RW3D was confirmed. Meanwhile, the accuracy of RWBayes was observed to have significantly higher Dice/VOE than the state-of-the-art interactive segmentation methods. To directly demonstrate the performance of our proposed method, in respect to the statistical significance analysis, the *p* value was the probability of obtaining a test statistic result that was actually observed. These statistical tests demonstrated that our proposed RWBayes approach yields the high precision results with respect to the conventional RW3D method (*p* < 0.001).

## 5. Discussion

This paper introduced a new knowledge-based framework for the organ segmentation using the RWBayes method. The proposed method segmented an organ based on a set of prior knowledge. Prior knowledge included the approximate shape of an organ (shape knowledge) and statistical parameters of the organ's intensities (intensity knowledge). According to a prior knowledge of an organ, the proper selection of object/background seeds was performed skillfully for our method to accurately segment the organ from the CT image.

The basic idea of the proposed method is based on the high correlation between adjacent slices. Seed points for the current slice are automatically generated according to the prior knowledge from the segmented organ region of the previous slice. As shown in Figure 5, precision results were achieved in our experiments as we used high-resolution data.

In practical clinics, however, CT images exist in various resolutions. In general, thin slices (high resolution) correspond to strong correlation while thick slices (low resolution) correspond to weak correlation.

**Figure 7:**
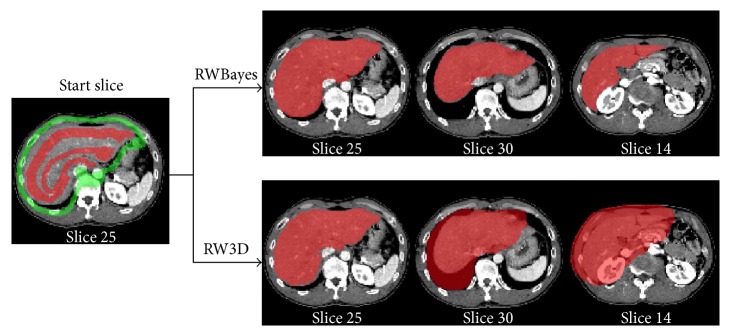
Effect of resolution on segmentation accuracy for case 1.

In order to verify the effect of resolutions on our RWBayes method, a typical CT image (a resolution of 0.683 × 0.683 × 1.25 mm^3^ and a size of 512 × 512 × 159 pixels) is resized into 7 different resolutions in the axial-axis (*z*-axis) and then segmented with the same seed points.  Figure 7 indicates that our proposed technique can be performed on the CT scans with large resolution variations. Regardless of image resolution, satisfactory segmentation results were achieved. In conclusion, our RWBayes method was robust in segmenting the livers from CT images of various resolutions. The simulation results prove the high capacity of our proposed RWBayes method for the organ segmentation using various resolutions of CT scans.

## 6. Conclusion

In this paper, we proposed a novel knowledge-based framework for organ segmentation using the RWBayes algorithm. A prior knowledge of the previous segmented organ was integrated into our strategy and has the following benefits: (1) small-scale graph; (2) automation of object/background seed setting according to the prior knowledge of the already segmented slices; and (3) robust segmentation technique by combing a Bayes model of an organ into the sparse system to calculate the probability of each unmarked node. The evaluation of the results demonstrated the high precision of the proposed approach. Compared with the conventional RW and the state-of-the-art interactive segmentation methods, our proposed method can significantly improve the segmentation accuracy (*p* < 0.001). As for future applications, the proposed method can be extended to segment other organs.

## Figures and Tables

**Table 1:** Segmentation accuracy obtained by the state-of-the-art methods for the liver on 26 CT scans.

	RW3D [37]	GC [34]	IKM [14]	RWNBT [44]	RWBayes
Dice	0.573	0.857	0.894	0.687	0.934
VOE	0.404	0.758	0.810	0.526	0.874
Runtime (sec)	45.800	1.828	2.530	1.781	1.231
